# Early Time-Restricted Feeding Improves 24-Hour Glucose Levels and Affects Markers of the Circadian Clock, Aging, and Autophagy in Humans

**DOI:** 10.3390/nu11061234

**Published:** 2019-05-30

**Authors:** Humaira Jamshed, Robbie A. Beyl, Deborah L. Della Manna, Eddy S. Yang, Eric Ravussin, Courtney M. Peterson

**Affiliations:** 1 Department of Nutrition Sciences, University of Alabama at Birmingham, Birmingham, AL 35294, USA; humaira.jphd@gmail.com; 2 Biostatistics and Analysis Laboratory, Pennington Biomedical Research Center, Baton Rouge, LA 70808, USA; robbie.beyl@pbrc.edu; 3 Department of Radiation Oncology, University of Alabama at Birmingham, Birmingham, AL 35294, USA; dellaman@uab.edu (D.L.D.M.); eyang@uab.edu (E.S.Y.); 4 Translational Physiology Laboratory, Pennington Biomedical Research Center, Baton Rouge, LA 70808, USA; eric.ravussin@pbrc.edu

**Keywords:** intermittent fasting, time-restricted feeding, meal timing, circadian rhythms, circadian system

## Abstract

Time-restricted feeding (TRF) is a form of intermittent fasting that involves having a longer daily fasting period. Preliminary studies report that TRF improves cardiometabolic health in rodents and humans. Here, we performed the first study to determine how TRF affects gene expression, circulating hormones, and diurnal patterns in cardiometabolic risk factors in humans. Eleven overweight adults participated in a 4-day randomized crossover study where they ate between 8 am and 2 pm (early TRF (eTRF)) and between 8 am and 8 pm (control schedule). Participants underwent continuous glucose monitoring, and blood was drawn to assess cardiometabolic risk factors, hormones, and gene expression in whole blood cells. Relative to the control schedule, eTRF decreased mean 24-hour glucose levels by 4 ± 1 mg/dl (*p* = 0.0003) and glycemic excursions by 12 ± 3 mg/dl (*p* = 0.001). In the morning before breakfast, eTRF increased ketones, cholesterol, and the expression of the stress response and aging gene *SIRT1* and the autophagy gene *LC3A* (all *p* < 0.04), while in the evening, it tended to increase brain-derived neurotropic factor (BNDF; *p* = 0.10) and also increased the expression of *MTOR* (*p* = 0.007), a major nutrient-sensing protein that regulates cell growth. eTRF also altered the diurnal patterns in cortisol and the expression of several circadian clock genes (*p* < 0.05). eTRF improves 24-hour glucose levels, alters lipid metabolism and circadian clock gene expression, and may also increase autophagy and have anti-aging effects in humans.

## 1. Introduction

Intermittent fasting (IF) covers a broad class of interventions that alternate periods of eating and extended fasting. IF interventions include periodic 24-hour fasts, intermittent energy restriction (e.g., the 5:2 diet), and time-restricted feeding. In animal models, IF has been found to improve cardiometabolic health, reduce cancer incidence, slow tumor growth, regenerate organs by increasing stem cell production, and increase lifespan [1,2]. In humans, data on IF is limited but suggest that it decreases body weight, insulin levels, blood pressure, inflammation, and appetite, and that it improves insulin sensitivity and lipid profiles [1,3,4,5]. These clinical benefits are driven by a reduction in insulin levels; improved insulin signaling; a reduction in oxidative stress; an increase in antioxidant defenses and autophagy; a reprogramming of aging-related pathways and hormones such as sirtuin 1 (SIRT1), brain-derived neurotrophic factor (BDNF), mechanistic target of rapamycin (mTOR), and insulin-like growth factor (IGF-1); and other mechanisms [6,7].

While the benefits of some types of IF may stem mostly or entirely from energy restriction [8,9], one form of IF, called time-restricted feeding (TRF), has demonstrated benefits independent of energy restriction in both animals [10,11,12,13,14,15,16,17] and humans [18,19]. Since the median American eats over a 12-hour period [20], we define TRF as eating within a ≤10-hour period and fasting for at least 14 hours per day. (Although TRF can include Ramadan fasting, we consider Ramadan fasting to be a separate type of IF.) Studies in rodents report that TRF reduces body weight, improves glycemic control, lowers insulin levels, reduces blood pressure, prevents hyperlipidemia, decreases hepatic fat, improves inflammatory markers, slows tumor growth, and increases lifespan, even when food intake is matched to the control group [10,11,12,13,14,15,16,17,21,22,23,24,25,26,27,28,29,30,31,32,33,34,35,36,37,38,39,40]. To date, there have been nine pilot-sized trials of TRF in humans [18,19,41,42,43,44,45,46,47]. Interestingly, TRF improved weight loss and cardiometabolic endpoints—such as insulin levels, insulin sensitivity, and blood pressure—when participants ate early or in the middle of the day [18,19,41,42,43,44,45], but worsened cardiometabolic health or had null effects when participants ate late in the day [46,47,48]. 

The circadian system may explain these dichotomous time-of-day effects. The circadian system orchestrates approximately 24-hour rhythms in metabolism, physiology, and behavior. It produces these rhythms through coordinated transcriptional–translational feedback loops involving clock genes such as *BMAL1, CLOCK, PER1/2*, and *CRY1/2,* which in turn cause oscillations in a myriad of downstream targets. For instance, insulin sensitivity and the thermic effect of food exhibit 24-hour rhythms, peaking in the morning [49]. A large number of plasma lipids [50] and age-related hormones such as cortisol, insulin, and growth hormone [51,52] also vary across the 24-hour day. Many of these metabolic and hormonal rhythms peak in the morning and are downregulated in the evening, implicating the morning as optimal for food intake [49]. Therefore, eating in sync with these rhythms may improve cardiometabolic health, as suggested by a growing number of human studies [53,54,55,56,57,58]. In contrast, eating in circadian misalignment with these rhythms by eating late in the day worsens several cardiometabolic endpoints, particularly glucose tolerance [59,60,61,62]. Therefore, TRF interventions where food intake is limited to early in the day may be particularly effective at improving cardiometabolic health.

We recently conducted the first clinical trials of early time-restricted feeding (eTRF), which combines the benefits of intermittent fasting with eating early in the day to be in sync with circadian rhythms in metabolism [18,19]. eTRF is tantamount to eating dinner in the mid-afternoon and fasting for the rest of the day. In our first 5-week crossover study, we found that eTRF reduces insulin levels, improves insulin sensitivity, lowers blood pressure, and decreases lipid peroxidation in men with prediabetes [18]. In our second 4-day crossover study, we investigated the effects of eTRF on energy metabolism in adults who are overweight and found that eTRF does not affect energy expenditure but increases fat oxidation, reduces the hunger hormone ghrelin, and improves subjective appetite [18,19]. Here, we extend our analyses from the 4-day trial to perform the first study of how TRF affects diurnal patterns in cardiometabolic risk factors, selected hormones, and the expression of glycemic and circadian clock genes in humans. As an exploratory aim, we also investigated the effects on the expression of genes related to aging, autophagy, and oxidative stress. We hypothesized that eTRF would decrease mean 24-hour glucose levels, positively impact hormones such as IGF-1 and BDNF, and alter circadian clock gene expression. 

## 2. Materials and Methods

### 2.1. Participants

This randomized controlled crossover study was approved by the Institutional Review Boards at Pennington Biomedical Research Center (PBRC; Baton Rouge, LA; protocol number: 2014-038) and the University of Alabama at Birmingham (Birmingham, AL; protocol number: 300001013). The study was pre-registered on ClinicalTrials.gov (NCT02247076) and conducted in accordance with the Declaration of Helsinki. 

Inclusion criteria targeted generally healthy adults aged 20–45 years old with a body mass index (BMI) between 25.0 kg and 35.0 kg/m^2^, a body weight between 68.0 kg and 100.0 kg, a regular bedtime between 21:30 and 24:00, and a regular menstrual cycle (if female). Individuals were excluded from the study if: they had diabetes or other significant chronic conditions; regularly used antidiabetic medications, steroids, beta blockers, adrenergic-stimulating agents, laxatives, or any medications or supplements known to affect sleep, circadian rhythms, or metabolism (with the exception of caffeine, which was allowed, except on the day of and prior to 24-hour testing); were pregnant or lactating; used Depo Provera, an IUD, or a hormonal patch for birth control; or had changed their hormonal birth control dose within the last 3 months. They were also excluded if they performed overnight shift work, had irregular sleep and/or eating schedules, regularly fasted for more than 15 hours/day, smoked or used nicotine/tobacco products within the last 3 months, consumed an average of more than 3 servings of alcohol per day, or regularly engaged in competitive sport training. All participants provided written informed consent and were provided a stipend for their participation.

### 2.2. Study Design

**Figure 1:**
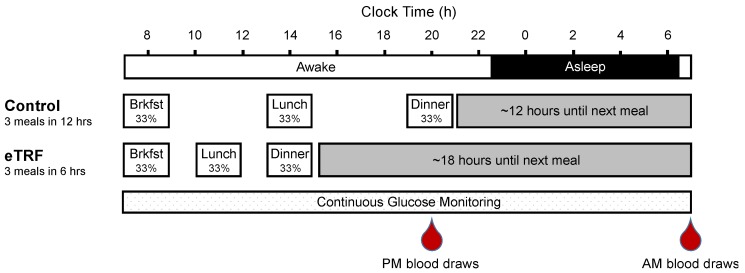
Study Protocol. Eleven participants were randomized to eat between 08:00 and 20:00 (control arm) or between 08:00 and 14:00 (early time-restricted feeding (eTRF) arm) for 4 days and then crossed over to the other arm after a 3.5–5-week washout period. On day 4, they consumed 3 identical meals that constituted one-third of their daily energy requirements, while undergoing 24-hour continuous glucose monitoring. In addition, blood was drawn in the evening (PM) on day 3 and in the morning (AM) on day 5 to measure serum analytes and gene expression.

This study was previously described in [19], and all Consolidated Standards of Reporting Trials (CONSORT) details can be found in the primary manuscript. In brief, participants were randomized to follow either the control schedule (eat between 8:00 and 20:00, a 12-hour eating period) or an eTRF schedule (eat between 08:00 and 14:00, a 6-hour eating period) for 4 days and then to crossover to the other arm after a 3.5–5-week washout period. Randomization was stratified by sex in blocks of 4. On days 1–2, participants followed their assigned eating schedule on their own and were instructed to maintain their habitual sleep and exercise habits. On days 3–4, participants continued to follow their assigned schedule but ate only food provided by study staff while under supervision. They also refrained from caffeine and exercise on days 3–4, to avoid influencing study outcomes. Breakfast, lunch, and dinner were served at 08:00, 14:00, and 20:00 for the control schedule, and at 08:00, 11:00, and 14:00 for the eTRF schedule (Figure 1). The three daily meals (50% carbohydrate, 35% fat, 15% protein) were matched across arms and were designed to meet weight-maintenance energy requirements under sedentary conditions, as described in [19]. No other food or beverages containing calories were allowed, and all food intake was matched across arms, with no weigh-backs allowed. On day 4, participants ate three identical meals, while completing a 24-hour stay in a respiratory chamber wearing a continuous glucose monitor. The mean physical activity level during the 24-hour testing was kept at a low value of 1.16 ± 0.10. Blood samples were collected in the fasting state at 20:00 on day 3 (evening, PM) and immediately after exiting the chamber at ~07:30 on day 5 (morning, AM). The evening blood draws were taken immediately before dinner in the control arm. Therefore, both arms fasted for 6 hours prior to the evening draws.

### 2.3. Continuous Glucose Monitoring

Participants wore a continuous glucose monitor (CGM; Dexcom G4, Platinum CGM System, San Diego, CA, USA) starting at approximately 08:30 on day 3 until 07:00 on day 5. The sensor was inserted under the skin into the subcutaneous fat of the abdomen, as per the manufacturer’s instructions, in the morning after breakfast on day 3. Participants returned for subsequent calibrations at 11:00 and 20:00 on day 3 and then approximately every 12 hours thereafter. Data from 07:00 on day 4 until 07:00 on day 5 were used to assess 24-hour glucose levels. Due to poor calibrations or nurse error, some data were clearly invalid. Data were deemed to be valid and were included in the analysis if the difference between the CGM and capillary measurements was less than 25 mg/dl for all calibrations in the 24-hour measurement period. In total, six data sets met this criterion. Occasional missing values (<0.06% of all data) were imputed by averaging adjacent values. Mean values were calculated for each 3-hour postprandial period, while participants were awake (06:30–20:30) and asleep (20:30–06:30), and for the whole 24-hour period. Each mean glucose value was calculated using the glucose area under the curve (AUC) divided by the length of the corresponding period. In the case of the 24-hour mean, we divided the 24-hour AUC value by 24 hours, which is equivalent to the estimated average glucose (eAG). Glycemic excursions were calculated using the Mean Amplitude of Glycemic Excursions (MAGE), which was tabulated using an Excel-enabled workbook called EasyGV [63]. 

### 2.4. Serum Chemistry

Cardiometabolic analytes and hormones were assessed in the fasting state in the morning and evening. Glucose, total cholesterol, and triglycerides were measured on a DXC600 instrument (Beckman Coulter, Inc.; Brea, CA, USA) using standard reagents. HDL cholesterol was measured using an immunoinhibition assay (Trinity Biotech USA, Inc.; Jamestown, NY, USA, or FUJIFILM Wako Chemicals USA Corporation; Richmond, CA, USA) on the same instrument. LDL cholesterol was calculated using the Friedewald equation. Insulin and cortisol were measured using chemiluminescent immunoassays on an Immulite 2000 instrument (Siemens Corporation; Washington, DC, USA). Homeostatic Model Assessment of Insulin Resistance (HOMA-IR), which is an estimate of insulin resistance, was calculated as fasting insulin (mU/l) × fasting glucose (mg/dl)/405. Free fatty acids (FFA) and β-hydroxybutyrate were run on a Sirrus Clinical Chemistry Analyzer (Stanbio Laboratory; Boerne, TX, USA), with the former measured using Wako reagents (FUJIFILM Wako Chemicals USA Corporation; Richmond, VA, USA). Human growth hormone (HGH) was run on an Automated Immunoassay Analyzer-990 (Tosoh Bioscience, Inc.; South San Francisco, CA, USA) using immunofluorescence. BDNF was run using an R&D Systems (Minneapolis, MN, USA) enzyme-linked immunosorbent assay (ELISA). IGF-1 and IGF-binding proteins 1 and 3 (IGFBP-1 and IGFBP-3) were run using American Laboratory Products Company (ALPCO; Salem, NH, USA) ELISAs. The manufacturer’s instructions were followed for all assays. 

### 2.5. Gene Expression

The expression of several genes related to glucose metabolism, the circadian system, fasting, autophagy, and oxidative stress was also assessed in the morning and evening. The full set of genes and their accession numbers are listed in Table S1 (Supplementary Materials). Whole blood was collected in Tempus™ Blood RNA tubes, and mRNA was later isolated from frozen samples using Tempus™ Spin RNA Isolation Kit (Applied Biosystems; Foster City, CA, USA). Extracted mRNA was included in the analysis if the concentration exceeded 55 ng/μl and the A260/A280 and A260/A230 ratios were between 2.12–2.26 and 2.07–3.21, respectively, as determined by a DeNovix DS-11 Spectrophotometer (DeNovix, Inc.; Wilmington, DE, USA) reading. These criteria were met at all four time points in eight of the 11 subjects. Samples were analyzed on the NanoString nCounter FLEX Analysis System (NanoString Technologies, Inc.; Seattle, WA, USA), following the manufacturer’s instructions. Data files were imported to the NanoString software nSolver 4.0, normalized using the geometric mean of six selected housekeeping genes (Table S1), and analyzed as fold changes.

### 2.6. Statistical Methods

Statistical significance was assessed per-protocol using two-sided tests with a type I error rate of α = 0.05. Treatment effects and *p*-values were calculated using a linear mixed model with heterogeneous compound symmetry in the software SAS (version 9.4; Cary, NC, USA). The Satterthwaite method was used for calculating the degrees of freedom, with participants serving as the random effect, and the treatment, sequence, period, and sex chosen as fixed effects. Treatment effects (Δ) are reported as least squares mean ± standard error of the mean (SEM). Data in the figures is displayed as raw mean ± SEM. Glycemic and circadian gene expression was a pre-specified outcome, while the expression of all other genes was considered to be exploratory and was adjusted for multiple comparisons using the Benjamini–Hochberg method, with a false discovery rate of 0.05.

## 3. Results

### 3.1. Participant Characteristics

Participant characteristics, the participant flow diagram, and adverse events were previously reported in [19]. In brief, 18 generally healthy participants were enrolled, and 11 participants completed both arms of the intervention. The 11 adults (7 men and 4 women; 64% African-American, 27% Caucasian, 9% Other) were aged 32 ± 7 years (mean ± standard deviation), had a mean BMI of 30.1 ± 2.7 kg/m^2^, and a mean fasting glucose of 92 ± 5 mg/dl.

### 3.2. 24-Hour Glucose Levels

**Figure 2:**
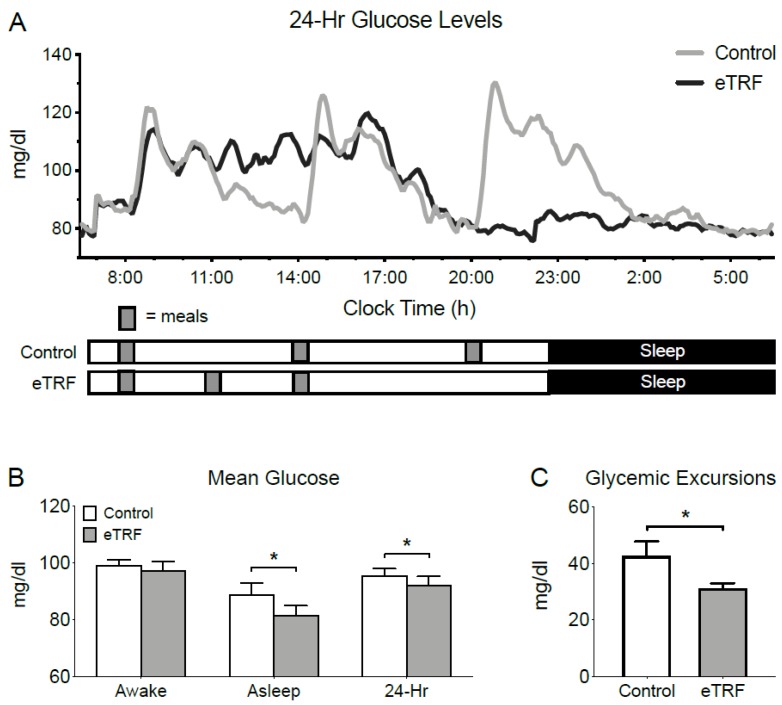
24-Hour Glucose Levels. Relative to the control schedule, early time-restricted feeding (eTRF) (**A**) changed the temporal profile of 24-hour glucose levels, as measured by continuous glucose monitoring, particularly in the evening, (**B**) lowered mean glucose levels while asleep and decreased 24-hour mean glucose levels, and (**C**) lowered glycemic excursions as measured by Mean Amplitude of Glycemic Excursions (MAGE). Error bars on panel (**A**) are suppressed for visual clarity. * *p* < 0.05.

As shown in Figure 2, eTRF changed temporal patterns in 24-hour glucose levels, as measured by CGM. Mean 3-hour postprandial glucose levels were 3 ± 1 mg/dl lower after breakfast (*p* = 0.05) but unchanged after lunch (2 ± 3 mg/dl; *p* = 0.51) and dinner (2 ± 3 mg/dl; *p* = 0.50) (data not shown). Although eTRF did not significantly affect mean glucose levels while participants were awake (06:30–22:30; *p* = 0.17), it lowered mean levels while they slept by 7 ± 2 mg/dl (22:30–6:30; *p* = 0.006). When aggregated across the day, eTRF reduced mean 24-hour glucose levels by 4 ± 1 mg/dl (*p* = 0.0003). eTRF also reduced glycemic excursions, as measured by MAGE, by 12 ± 3 mg/dl (*p* = 0.001). 

### 3.3. Glycemic Markers

**Figure 3:**
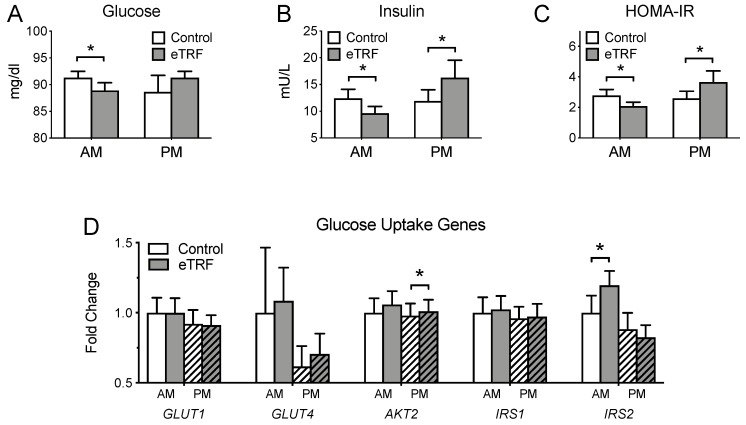
Glycemic Markers. Relative to the control schedule, early time-restricted feeding (eTRF) decreased (**A**) fasting glucose, (**B**) fasting insulin, and (**C**) Homeostatic Model Assessment of Insulin Resistance (HOMA-IR) in the morning (AM) and increased (**B**) fasting insulin and (**C**) HOMA-IR in the evening (PM). (**D**) eTRF also increased the expression of the *IRS2* and *AKT2* genes in the morning and evening, respectively. Data for *GLUT1* and *GLUT4* are shown excluding a participant whose expression levels were ~10× higher than the sample mean. * *p* < 0.05.

Figure 3 shows the fasting values of glycemic markers in both the morning and evening. In the morning, eTRF lowered fasting glucose and insulin by 2 ± 1 mg/dl (*p* = 0.02) and 2.9 ± 0.4 mU/l (*p* < 0.0001), respectively. As a result, HOMA-IR was lower by 0.73 ± 0.11 (*p* < 0.0001). This was accompanied by a 25 ± 9% increase in *IRS2* gene expression (*p* = 0.01). In the evening, eTRF increased fasting insulin and HOMA-IR by 4.5 ± 1.6 mU/l (*p* = 0.01) and 1.09 ± 0.43 (*p* = 0.02), respectively, but did not affect glucose levels (*p* = 0.30). This was accompanied by a 4 ± 1% increase in *AKT2* gene expression (*p* = 0.003). There were no changes in *GLUT1*, *GLUT4,* or *IRS1* expression at either time of day (*p* ≥ 0.46). This was true regardless of whether or not a single outlier with ~10× higher *GLUT1* and *GLUT4* expression was included in the analyses.

### 3.4. Lipids

**Figure 4:**
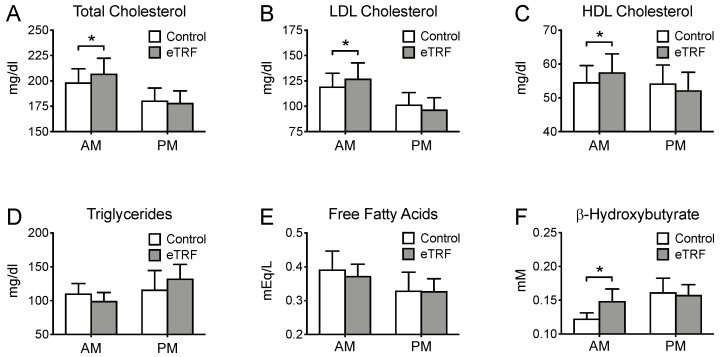
Lipids. Relative to the control arm, early time-restricted feeding (eTRF) increased fasting levels of (**A**) total cholesterol, (**B**) LDL cholesterol, (**C**) HDL cholesterol, and (**F**) β-hydroxybutyrate (ketones) in the morning (AM) but did not affect levels of (**D**) triglycerides or (**E**) free fatty acids. (**A**–**F**). Levels of all lipids were unaffected in the evening (PM). * *p* < 0.05.

Figure 4 displays the fasting values of lipids in both the morning and evening. In the morning, eTRF increased LDL and HDL cholesterol by 9 ± 4 mg/dl (*p* = 0.02) and 3 ± 1 mg/dl (*p* = 0.03), respectively, but did not affect levels of triglycerides (*p* = 0.29) or free fatty acids (*p* = 0.73). As a result, total cholesterol was elevated by 10 ± 4 mg/dl (*p* = 0.04). eTRF also increased morning ketone levels, as measured by β-hydroxybutyrate, by 0.03 ± 0.01 mM (*p* = 0.009), causing them to reach a plasma concentration of 0.15 ± 0.6 mM. By contrast, in the evening, there were no differences in any lipid levels (*p* ≥ 0.18). There were also no differences in the HDL/LDL cholesterol ratio in either the morning or evening (*p* ≥ 0.54).

### 3.5. Hormones

**Figure 5:**
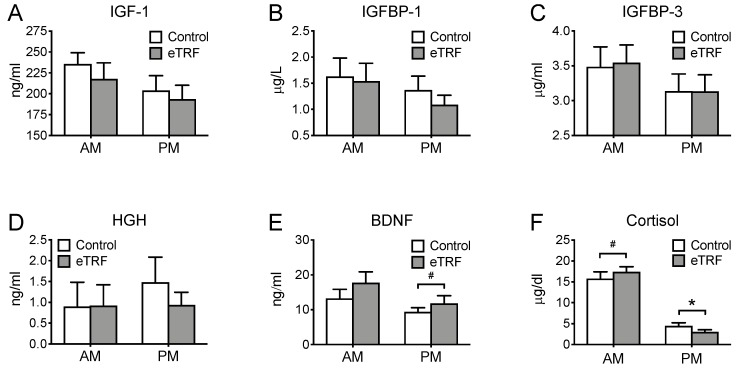
Hormones. Relative to the control arm, early time-restricted feeding (eTRF) tended to increase (**F**) cortisol levels in the morning (AM). In the evening, it lowered (**F**) cortisol and tended to increase (**E**) brain-derived neurotrophic factor (BDNF). The hormones (**A**) insulin-like growth factor (IGF-1), (**B**) IGF-binding protein 1 (IGFBP-1), (**C**) IGF-binding protein 3 (IGFBP-3), and (**D**) human growth hormone (HGH) were not significantly different between arms. * *p* < 0.05, ^#^ *p* < 0.10.

We also investigated the effects of meal timing on selected hormones that are thought to be responsive to and/or mediate the beneficial effects of fasting (Figure 5). eTRF did not affect any of the hormones in the morning (*p* ≥ 0.26), except cortisol, which tended to increase by 1.5 ± 0.9 μg/dl (*p* = 0.10). In the evening, eTRF reduced cortisol levels by 1.4 ± 0.6 μg/dl (*p* = 0.03) and tended to increase BDNF levels by 2.46 ± 1.34 ng/ml (*p* = 0.09). Apparent decreases in evening levels of IGF-1 and IGFBP-1 did not reach statistical significance (both *p* = 0.11), while IGFBP-3 and HGH levels were unchanged (*p* ≥ 0.25).

### 3.6. Gene Expression

**Figure 6:**
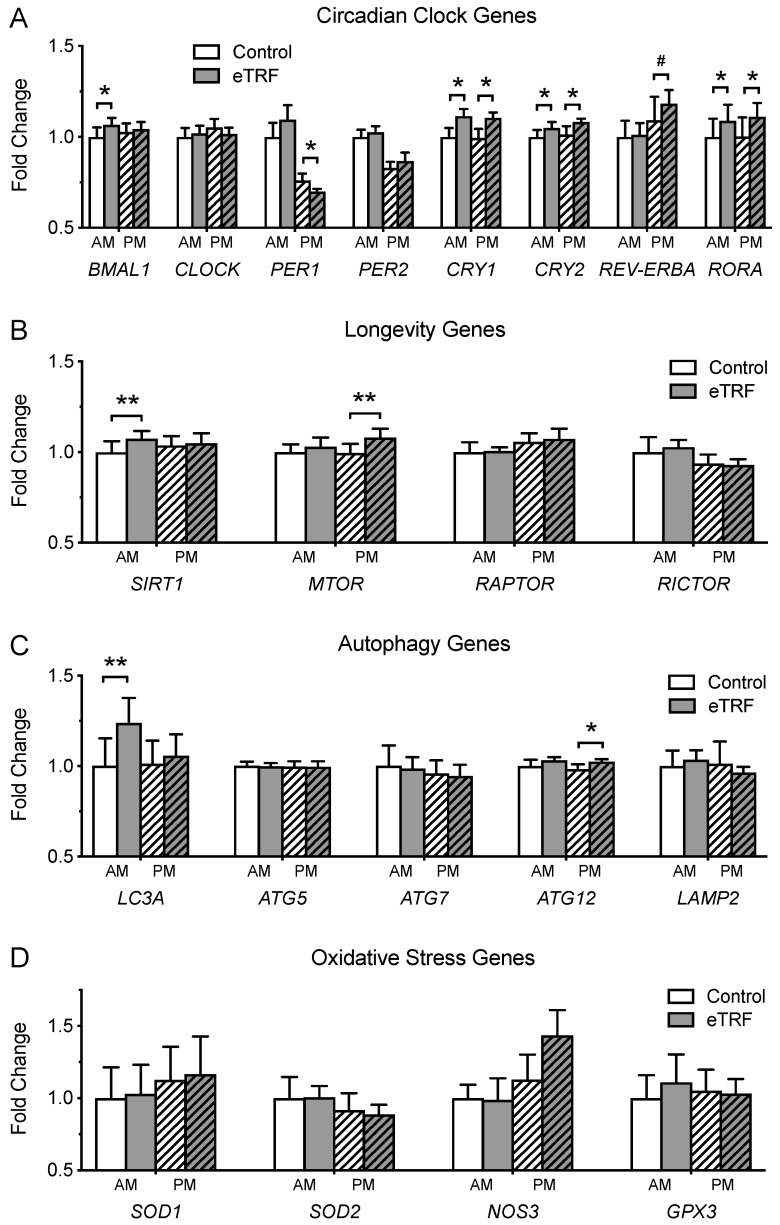
Gene Expression in Whole Blood. (**A**) Early time-restricted feeding (eTRF) changed the expression of several circadian clock genes, including *BMAL1*, *PER1*, *CRY1*, *CRY2*, *REV-ERBA,* and *RORA* in the morning (AM) and/or evening (PM). It also increased the expression of (**B**) the longevity genes *SIRT1* in the morning and *MTOR* in the evening. (**C**) The autophagy genes *LC3A* and *ATG12* were elevated in the morning and evening, respectively, although the latter was no longer significant after adjustment for multiple comparisons. (**D**) The expression of oxidative stress genes was unaffected. Genes in panel **A** were pre-specified outcomes, while genes in panels **B**–**D** were exploratory and had their *p*-values adjusted for multiple comparisons. ^#^ *p* < 0.10 (applied only to pre-specified genes in panel **A**), * *p* < 0.05, ** *p* < 0.007 (significant after adjustment for multiple comparisons, applied only to exploratory genes in panels **B**–**D**).

Figure 6 illustrates the effects of meal timing on the expression of genes related to (A) the circadian clock, (B) longevity, (C) autophagy, and (D) oxidative stress. eTRF significantly increased the expression of the circadian clock genes *BMAL1* (8 ± 3%; *p* = 0.007), *CRY1* (14 ± 2%; *p* < 0.0001), *CRY2* (8 ± 4%; *p* = 0.02), and *RORA* (12 ± 4%; *p* = 0.003) in the morning. In the evening, it decreased levels of *PER1* (−10 ± 4%; *p* = 0.02) and increased (or tended to increase) levels of *CRY1* (14 ± 4%; *p* = 0.006), *CRY2* (8 ± 4%; *p* = 0.05), *REV-ERBA* (12 ± 6%; *p* = 0.08), and *RORA* (13 ± 4%; *p* = 0.006). *CLOCK* and *PER2* were unaffected at both times of day (p ≥ 0.32). eTRF also increased levels of *SIRT1* (10 ± 3%; *p* = 0.004) and *LC3A* (22 ± 5%; *p* = 0.001) in the morning and increased levels of *MTOR* (9 ± 3%; *p* = 0.007) in the evening. The autophagy gene *ATG12* was elevated (5 ± 2%; *p* = 0.04) in the evening, but this effect was no longer significant after adjustment for multiple comparisons. The expression of all other genes was unchanged (*p* ≥ 0.13). 

## 4. Discussion

Time-restricted feeding (TRF) is a novel form of intermittent fasting that improves cardiometabolic health, slows tumor progression, delays aging, and increases lifespan in rodents [10,11,12,13,14,15,16,17,21,22,23,24,25,26,27,28,29,30,31,32,33,34,35,36,37,38,39,40]. Pilot studies in humans similarly suggest that TRF improves clinical outcomes such as body weight, blood pressure, and insulin sensitivity [18,19,41,42,43,44,45,46,47], at least when food intake is limited to early or the middle of the day. These time-of-day effects may be explained by the circadian system, as eating in alignment with circadian rhythms in metabolism appears to improve cardiometabolic health [53,54,55,56,57,58]. However, the molecular mechanisms underlying TRF in humans were unknown. Here, to our knowledge and excluding studies on Ramadan fasting, we performed the first clinical trial to determine how TRF affects gene expression and diurnal patterns in cardiometabolic risk factors in humans. Our study is the first investigation into the molecular mechanisms underlying TRF in humans, as well as the second clinical trial of early time-restricted feeding (eTRF) in humans. 

Relative to the control schedule, eTRF decreased mean 24-hour levels of glucose. More than two dozen studies have previously reported diurnal rhythms in glycemic control, with glucose tolerance peaking in the morning [49]. The circadian system causes both insulin sensitivity and first-phase insulin secretion to be upregulated in the morning, and human studies report that the incremental glucose AUC is up to two-fold higher in the evening relative to the morning [64]. Since glucose tolerance is highest in the morning, we expected that shifting a majority of daily food intake to the morning would decrease mean 24-hour glucose levels. Indeed, the largest temporal differences in plasma glucose were observed in the late evening and while sleeping. In fact, in the control arm, glucose levels remained elevated during nearly half of the sleep episode. Given that the control arm was designed to represent median adult eating times in the US, eating dinner at 8 pm leads to a prolonged elevation of glucose levels while asleep and may have adverse metabolic consequences, such as impairing fat oxidation [65].

In addition to lowering mean 24-hour glucose levels, eTRF also lowered fasting glucose and insulin in the morning, increased fasting insulin in the evening, and decreased 24-hour glycemic excursions. The decreased glucose and insulin in the morning were accompanied by an increase in *AKT2* expression. Since the protein Akt2 is a downstream target of insulin signaling via the phosphatidylinositol 3-kinase (PI3K) pathway and plays a key role in insulin-stimulated glucose uptake, this suggests that eTRF may improve insulin signaling in the morning. This is consistent with data from our previous trial showing that 5 weeks of eTRF reduced insulin levels and improved insulin sensitivity during an oral glucose tolerance test administered in the morning [18]. Although we are not certain how to interpret the evening increases in fasting insulin and expression of the insulin signaling protein *IRS2* (an insulin receptor substrate that modulates mitogenic and anti-apoptotic signaling pathways) in the eTRF arm, we note that they may be reflective of differences in cumulative energy intake at that time point during the day. The decrease in glycemic excursions was somewhat contrary to our expectations. We would have expected peak postprandial glucose levels to be higher in the eTRF arm since meals were eaten in short succession. However, we found the opposite to be true. We speculate that one possible explanation for the decrease in peak glucose levels, particularly at lunchtime, may be that circulating insulin levels were still elevated because breakfast was still being digested and therefore the *β*-cells in the pancreas did not have to be “re-awakened” to secrete insulin, eliminating the lag time between rising glucose and insulin levels and thereby lessening any spikes in plasma glucose. Importantly, our data suggest that TRF and any other approach where meals are eaten in short succession before the prior meal is fully digested may lower glycemic excursions—conferring additional glycemic benefits through mechanisms independent of the circadian system. This suggests that TRF interventions where the inter-meal interval is short may be particularly effective at improving 24-hour glucose levels. Conversely, we speculate that TRF interventions where meals are eaten too far apart (e.g., more than 4–5 hours apart), such as when the daily eating period is longer than 8–10 hours and/or involves only 2 meals/day, may be less effective at improving 24-hour glucose levels. This underscores the fact that while IF interventions are often viewed as synonymous with a reduction in meal frequency, practicing IF and reducing meal frequency are not the same thing, and future studies on IF should investigate whether the inter-meal interval and meal frequency influence health outcomes.

Lipids and hormones were also affected by meal timing. eTRF increased LDL and HDL cholesterol in the morning, which may be attributed to the prolonged fasting period and greater reliance on fat oxidation in the eTRF arm [19]. It will be important to confirm in future studies that the slight increase in both LDL and HDL cholesterol in the morning is not pathophysiologic. However, we did not observe any increases in triglycerides and free fatty acids, as would be expected from the extended fasting. These results are similar to those reported in [42] but contrast with those reported in [18]. The reasons for the latter dissimilarity are unclear but may be due to differences in the intervention duration, the study population, or other factors. eTRF also increased β-hydroxybutyrate in the morning, relative to the control arm, thus demonstrating that even short-term daily fasting can modestly increase circulating ketones. Elevated ketone levels reduce oxidative stress, preserve lean mass [66], and have other metabolic effects such as decreasing hunger, although it is unclear whether the modest changes that we observed would be clinically significant. Among the hormonal endpoints, cortisol, which is a metabolic and circadian hormone, tended to increase in the morning and decrease in the evening. This suggests that eTRF may have increased the amplitude of the cortisol rhythm, providing a mechanism through which meal timing may impact the circadian system. Therefore, contrary to widespread belief, meal timing may directly impact the central circadian clock. Of the growth-related hormones, eTRF tended to elevate BDNF levels in the evening. BNDF promotes neuronal growth, development, and survival and is widely known to be increased by intermittent fasting in rodents [6]. Our study is one of the first trials to demonstrate that intermittent fasting can increase BDNF levels in humans. Although we additionally expected to observe a decrease in IGF-1, which is associated with cancer risk and aging, declines in IGF-1 and IGFBP-1 in the evening did not quite reach statistical significance (both *p* = 0.11).

We also measured diurnal changes in gene expression. Only 4 days of eTRF surprisingly induced wide-sweeping changes in circadian clock gene expression, with 6 out of 8 circadian genes affected. Our data are corroborated by a trial reporting that a single bout of breakfast skipping changes the postprandial expression of several clock genes in whole blood [67], suggesting that a tightly-controlled, bidirectional feedback loop exists between meal timing and the circadian system. We also detected changes in several exploratory gene targets. Both *SIRT1* and *LC3A* were upregulated in the morning before breakfast, while *MTOR* was upregulated in the evening. mTOR is a nutrient-sensing phosphatidylinositol 3-kinase-related kinase, which is stimulated by insulin, protein, and growth factors to drive protein synthesis and regulate cell growth, differentiation, and metabolism. Its observed upregulation in the evening likely mirrored the increase in fasting insulin. SIRT1 is nicotinamide adenosine dinucleotide (NAD)-dependent deacetylase that promotes insulin secretion and action; upregulates fat metabolism; protects against inflammation, oxidative stress, and DNA damage; increases telomere stability; and extends lifespan [68]. The increase in *SIRT1* expression in the morning suggests that eTRF may also promote longevity in humans, as it does in animals [34]. A recent study in rodents found that at least 40% of the lifespan-extending effects of caloric restriction could instead be attributed to TRF [34]. Lastly, eTRF increased *LC3A* expression by 22% in the morning at the end of the 18-hour fast. *LC3A* encodes an essential structural component of autophagosomal membranes, and autophagy has been shown to play a major role in protecting against multiple chronic diseases such as diabetes, heart disease, cancer, and neurodegenerative diseases, by recycling damaged and used proteins and organelles. Increasing autophagy may have anti-aging or rejuvenating effects. Although no previous studies examining TRF as a meal timing intervention have investigated autophagy in either animals or humans, other studies on intermittent fasting conclude that several of the benefits of intermittent fasting are mediated through enhanced autophagy [69,70]. By comparison, we observed no changes in the expression of the four antioxidant genes measured.

This study has several limitations. Although our study was highly rigorous in design, the sample size was small, and several of our endpoints were likely underpowered. For instance, substantial decreases in IGF-1 and IGFBP-1 (both *p* = 0.11) and increases in *NOS3* gene expression (*p* = 0.13), which would provide further evidence that eTRF may slow aging and reduce cancer risk, did not reach statistical significance. Thus, our null findings for serum analytes and genes should be interpreted with caution, and these targets need to be re-tested in larger trials. Similarly, because some of our CGM data was not well-calibrated and had to be excluded, resulting in a smaller sample size, such favorable data should also be viewed more cautiously. Although, in this case, prior studies do concur that shifting food intake to early in the day improves mean daily glucose levels [42,54]. Second, our study intervention was only 4 days, which may be insufficient for circadian and/or metabolic adaptation to occur. Third, with the exception of glucose, all endpoints were measured at only two times of day. We lack data on endpoints in the postprandial state and also while sleeping. Because some endpoints, such as autophagy, are likely maximally upregulated during sleep, we may have missed detecting or underestimated the effect sizes of diurnal changes in several endpoints. Also, although the PM blood draws were taken after a 6-hour fast in both arms, there were differences in cumulative food intake between the study arms, which may have impacted the endpoints measured in the evening. In our trial, we were not able to draw blood at multiple time points throughout the day while our participants resided in the respiratory chamber, but future studies would benefit from measuring these endpoints across the 24-hour day. Lastly, gene expression data are known to be limited in nature and not necessarily reflective of changes in protein levels or activation.

Collectively, our data suggest that eTRF improves several facets of health through both circadian- and fasting-related mechanisms. eTRF improves glycemic control by lowering 24-hour glucose levels, reducing glycemic excursions, and potentially by improving insulin signaling. Importantly, some of these improvements in glycemic excursions may be driven not only by eating earlier in the day but also by having a short inter-meal interval, suggesting that TRF interventions with longer inter-meal intervals may be less effective at improving glucose levels. We also found that eTRF alters diurnal patterns in fasting cholesterol, ketones, cortisol, and circadian clock genes; in particular, it modestly increases ketone levels in the morning and improves the amplitude of the cortisol rhythm. Finally, eTRF favorably affects hormones and genes related to longevity and autophagy such as BDNF, *SIRT1,* and *LC3A*. These important findings demonstrate that eTRF improves cardiometabolic health, alters diurnal rhythms, and may have anti-aging effects. Further research in humans is needed to replicate and extend these results.

## Supplementary Materials

The following are available online at https://www.mdpi.com/2072-6643/11/6/1234/s1, Table S1: Genes and Their Accession Numbers.

## References

[1] Mattson M.P., Longo V.D., Harvie M. (2017). Impact of intermittent fasting on health and disease processes. Ageing Res. Rev..

[2] Harvie M., Howell A. (2017). Potential Benefits and Harms of Intermittent Energy Restriction and Intermittent Fasting Amongst Obese, Overweight and Normal Weight Subjects-A Narrative Review of Human and Animal Evidence. Behav. Sci..

[3] Tinsley G.M., Horne B.D. (2018). Intermittent fasting and cardiovascular disease: Current evidence and unresolved questions. Fut. Cardiol..

[4] Patterson R.E., Sears D.D. (2017). Metabolic Effects of Intermittent Fasting. Annu. Rev. Nutr..

[5] Antoni R., Johnston K.L., Collins A.L., Robertson M.D. (2017). Effects of intermittent fasting on glucose and lipid metabolism. Proc. Nutr. Soc..

[6] Longo V.D., Mattson M.P. (2014). Fasting: Molecular mechanisms and clinical applications. Cell Metab..

[7] Anton S.D., Moehl K., Donahoo W.T., Marosi K., Lee S.A., Mainous A.G., Leeuwenburgh C., Mattson M.P. (2018). Flipping the Metabolic Switch: Understanding and Applying the Health Benefits of Fasting. Obesity.

[8] Cioffi I., Evangelista A., Ponzo V., Ciccone G., Soldati L., Santarpia L., Contaldo F., Pasanisi F., Ghigo E., Bo S. (2018). Intermittent versus continuous energy restriction on weight loss and cardiometabolic outcomes: A systematic review and meta-analysis of randomized controlled trials. J. Transl. Med..

[9] Seimon R.V., Roekenes J.A., Zibellini J., Zhu B., Gibson A.A., Hills A.P., Wood R.E., King N.A., Byrne N.M., Sainsbury A. (2015). Do intermittent diets provide physiological benefits over continuous diets for weight loss? A systematic review of clinical trials. Mol. Cell Endocrinol..

[10] Hatori M., Vollmers C., Zarrinpar A., DiTacchio L., Bushong E.A., Gill S., Leblanc M., Chaix A., Joens M., Fitzpatrick J.A. (2012). Time-restricted feeding without reducing caloric intake prevents metabolic diseases in mice fed a high-fat diet. Cell Metab..

[11] Zarrinpar A., Chaix A., Yooseph S., Panda S. (2014). Diet and feeding pattern affect the diurnal dynamics of the gut microbiome. Cell Metab..

[12] Sherman H., Genzer Y., Cohen R., Chapnik N., Madar Z., Froy O. (2012). Timed high-fat diet resets circadian metabolism and prevents obesity. FASEB J..

[13] Wu T., Sun L., ZhuGe F., Guo X., Zhao Z., Tang R., Chen Q., Chen L., Kato H., Fu Z. (2011). Differential roles of breakfast and supper in rats of a daily three-meal schedule upon circadian regulation and physiology. Chronobiol. Int..

[14] Olsen M.K., Choi M.H., Kulseng B., Zhao C.M., Chen D. (2017). Time-restricted feeding on weekdays restricts weight gain: A study using rat models of high-fat diet-induced obesity. Physiol. Behav..

[15] Chaix A., Lin T., Le H.D., Chang M.W., Panda S. (2019). Time-Restricted Feeding Prevents Obesity and Metabolic Syndrome in Mice Lacking a Circadian Clock. Cell Metab..

[16] Cote I., Toklu H.Z., Green S.M., Morgan D., Carter C.S., Tumer N., Scarpace P.J. (2018). Limiting feeding to the active phase reduces blood pressure without the necessity of caloric reduction or fat mass loss. Am. J. Physiol. Regul. Integr. Comp. Physiol..

[17] Delahaye L.B., Bloomer R.J., Butawan M.B., Wyman J.M., Hill J.L., Lee H.W., Liu A.C., McAllan L., Han J.C., van der Merwe M. (2018). Time-restricted feeding of a high-fat diet in male C57BL/6 mice reduces adiposity but does not protect against increased systemic inflammation. Appl. Physiol. Nutr. Metab..

[18] Sutton E.F., Beyl R., Early K.S., Cefalu W.T., Ravussin E., Peterson C.M. (2018). Early Time-Restricted Feeding Improves Insulin Sensitivity, Blood Pressure, and Oxidative Stress Even without Weight Loss in Men with Prediabetes. Cell Metab..

[19] Ravussin E., Beyl R.A., Poggiogalle E., Hsia D.S., Peterson C.M. (2019). Early Time-Restricted Feeding Reduces Appetite and Increases Fat Oxidation but Does Not Affect Energy Expenditure in Humans. Obesity.

[20] Kant A.K., Graubard B.I. (2014). Association of self-reported sleep duration with eating behaviors of American adults: NHANES 2005–2010. Am. J. Clin. Nutr..

[21] Belkacemi L., Selselet-Attou G., Louchami K., Sener A., Malaisse W.J. (2010). Intermittent fasting modulation of the diabetic syndrome in sand rats. II. In vivo investigations. Int. J. Mol. Med..

[22] Sherman H., Frumin I., Gutman R., Chapnik N., Lorentz A., Meylan J., le Coutre J., Froy O. (2011). Long-term restricted feeding alters circadian expression and reduces the level of inflammatory and disease markers. J. Cell. Mol. Med..

[23] Belkacemi L., Selselet-Attou G., Bulur N., Louchami K., Sener A., Malaisse W.J. (2011). Intermittent fasting modulation of the diabetic syndrome in sand rats. III. Post-mortem investigations. Int. J. Mol. Med..

[24] Duncan M.J., Smith J.T., Narbaiza J., Mueez F., Bustle L.B., Qureshi S., Fieseler C., Legan S.J. (2016). Restricting feeding to the active phase in middle-aged mice attenuates adverse metabolic effects of a high-fat diet. Physiol. Behav..

[25] Sundaram S., Yan L. (2016). Time-restricted feeding reduces adiposity in mice fed a high-fat diet. Nutr. Res..

[26] Chung H., Chou W., Sears D.D., Patterson R.E., Webster N.J., Ellies L.G. (2016). Time-restricted feeding improves insulin resistance and hepatic steatosis in a mouse model of postmenopausal obesity. Metabolism.

[27] Philippens K.M., von Mayersbach H., Scheving L.E. (1977). Effects of the scheduling of meal-feeding at different phases of the circadian system in rats. J. Nutr..

[28] Kudo T., Akiyama M., Kuriyama K., Sudo M., Moriya T., Shibata S. (2004). Night-time restricted feeding normalises clock genes and Pai-1 gene expression in the db/db mouse liver. Diabetologia.

[29] Manzanero S., Erion J.R., Santro T., Steyn F.J., Chen C., Arumugam T.V., Stranahan A.M. (2014). Intermittent fasting attenuates increases in neurogenesis after ischemia and reperfusion and improves recovery. J. Cereb. Blood Flow Metab..

[30] Garcia-Luna C., Soberanes-Chavez P., de Gortari P. (2017). Prepuberal light phase feeding induces neuroendocrine alterations in adult rats. J. Endocrinol..

[31] Park S., Yoo K.M., Hyun J.S., Kang S. (2017). Intermittent fasting reduces body fat but exacerbates hepatic insulin resistance in young rats regardless of high protein and fat diets. J. Nutr. Biochem..

[32] Belkacemi L., Selselet-Attou G., Hupkens E., Nguidjoe E., Louchami K., Sener A., Malaisse W.J. (2012). Intermittent fasting modulation of the diabetic syndrome in streptozotocin-injected rats. Int. J. Endocrinol..

[33] Chaix A., Zarrinpar A., Miu P., Panda S. (2014). Time-restricted feeding is a preventative and therapeutic intervention against diverse nutritional challenges. Cell Metab..

[34] Mitchell S.J., Bernier M., Mattison J.A., Aon M.A., Kaiser T.A., Anson R.M., Ikeno Y., Anderson R.M., Ingram D.K., de Cabo R. (2019). Daily Fasting Improves Health and Survival in Male Mice Independent of Diet Composition and Calories. Cell Metab..

[35] Smith N.J., Caldwell J.L., van der Merwe M., Sharma S., Butawan M., Puppa M., Bloomer R.J. (2019). A Comparison of Dietary and Caloric Restriction Models on Body Composition, Physical Performance, and Metabolic Health in Young Mice. Nutrients.

[36] Sun S., Hanzawa F., Umeki M., Ikeda S., Mochizuki S., Oda H. (2018). Time-restricted feeding suppresses excess sucrose-induced plasma and liver lipid accumulation in rats. PLoS ONE.

[37] Woodie L.N., Luo Y., Wayne M.J., Graff E.C., Ahmed B., O’Neill A.M., Greene M.W. (2018). Restricted feeding for 9h in the active period partially abrogates the detrimental metabolic effects of a Western diet with liquid sugar consumption in mice. Metabolism.

[38] Sundaram S., Yan L. (2018). Time-restricted feeding mitigates high-fat diet-enhanced mammary tumorigenesis in MMTV-PyMT mice. Nutr. Res..

[39] Li X.M., Delaunay F., Dulong S., Claustrat B., Zampera S., Fujii Y., Teboul M., Beau J., Levi F. (2010). Cancer inhibition through circadian reprogramming of tumor transcriptome with meal timing. Cancer Res..

[40] Wu M.W., Li X.M., Xian L.J., Levi F. (2004). Effects of meal timing on tumor progression in mice. Life Sci..

[41] Gabel K., Hoddy K.K., Haggerty N., Song J., Kroeger C.M., Trepanowski J.F., Panda S., Varady K.A. (2018). Effects of 8-hour time restricted feeding on body weight and metabolic disease risk factors in obese adults: A pilot study. Nutr. Healthy Aging.

[42] Antoni R., Robertson T.M., Robertson M., Johnston J. (2018). A pilot feasibility study exploring the effects of a moderate time-restricted feeding intervention on energy intake, adiposity and metabolic physiology in free-living human subjects. J. Nutr. Sci..

[43] Gasmi M., Sellami M., Denham J., Padulo J., Kuvacic G., Selmi W., Khalifa R. (2018). Time-restricted feeding influences immune responses without compromising muscle performance in older men. Nutrition.

[44] Moro T., Tinsley G., Bianco A., Marcolin G., Pacelli Q.F., Battaglia G., Palma A., Gentil P., Neri M., Paoli A. (2016). Effects of eight weeks of time-restricted feeding (16/8) on basal metabolism, maximal strength, body composition, inflammation, and cardiovascular risk factors in resistance-trained males. J. Transl. Med..

[45] Gill S., Panda S. (2015). A Smartphone App Reveals Erratic Diurnal Eating Patterns in Humans that Can Be Modulated for Health Benefits. Cell Metab..

[46] Tinsley G.M., Forsse J.S., Butler N.K., Paoli A., Bane A.A., La Bounty P.M., Morgan G.B., Grandjean P.W. (2017). Time-restricted feeding in young men performing resistance training: A randomized controlled trial. Eur. J. Sport Sci..

[47] Stote K.S., Baer D.J., Spears K., Paul D.R., Harris G.K., Rumpler W.V., Strycula P., Najjar S.S., Ferrucci L., Ingram D.K. (2007). A controlled trial of reduced meal frequency without caloric restriction in healthy, normal-weight, middle-aged adults. Am. J. Clin. Nutr..

[48] Carlson O., Martin B., Stote K.S., Golden E., Maudsley S., Najjar S.S., Ferrucci L., Ingram D.K., Longo D.L., Rumpler W.V. (2007). Impact of reduced meal frequency without caloric restriction on glucose regulation in healthy, normal-weight middle-aged men and women. Metabolism.

[49] Poggiogalle E., Jamshed H., Peterson C.M. (2018). Circadian regulation of glucose, lipid, and energy metabolism in humans. Metabolism.

[50] Dallmann R., Viola A.U., Tarokh L., Cajochen C., Brown S.A. (2012). The human circadian metabolome. Proc. Natl. Acad. Sci. USA.

[51] Gamble K.L., Berry R., Frank S.J., Young M.E. (2014). Circadian clock control of endocrine factors. Nat. Rev. Endocrinol..

[52] Tsang A.H., Astiz M., Friedrichs M., Oster H. (2016). Endocrine regulation of circadian physiology. J. Endocrinol..

[53] Rabinovitz H.R., Boaz M., Ganz T., Jakubowicz D., Matas Z., Madar Z., Wainstein J. (2014). Big breakfast rich in protein and fat improves glycemic control in type 2 diabetics. Obesity.

[54] Jakubowicz D., Barnea M., Wainstein J., Froy O. (2013). High caloric intake at breakfast vs. dinner differentially influences weight loss of overweight and obese women. Obesity.

[55] Reid K.J., Baron K.G., Zee P.C. (2014). Meal timing influences daily caloric intake in healthy adults. Nutr. Res..

[56] Ruiz-Lozano T., Vidal J., de Hollanda A., Scheer F.A., Garaulet M., Izquierdo-Pulido M. (2016). Timing of food intake is associated with weight loss evolution in severe obese patients after bariatric surgery. Clin. Nutr..

[57] Garaulet M., Gomez-Abellan P., Alburquerque-Bejar J.J., Lee Y.C., Ordovas J.M., Scheer F.A. (2013). Timing of food intake predicts weight loss effectiveness. Int. J. Obes..

[58] Keim N.L., Van Loan M.D., Horn W.F., Barbieri T.F., Mayclin P.L. (1997). Weight loss is greater with consumption of large morning meals and fat-free mass is preserved with large evening meals in women on a controlled weight reduction regimen. J. Nutr..

[59] Scheer F.A., Hilton M.F., Mantzoros C.S., Shea S.A. (2009). Adverse metabolic and cardiovascular consequences of circadian misalignment. Proc. Natl. Acad. Sci. USA.

[60] Wefers J., van Moorsel D., Hansen J., Connell N.J., Havekes B., Hoeks J., van Marken Lichtenbelt W.D., Duez H., Phielix E., Kalsbeek A. (2018). Circadian misalignment induces fatty acid metabolism gene profiles and compromises insulin sensitivity in human skeletal muscle. Proc. Natl. Acad. Sci. USA.

[61] Morris C.J., Purvis T.E., Hu K., Scheer F.A. (2016). Circadian misalignment increases cardiovascular disease risk factors in humans. Proc. Natl. Acad. Sci. USA.

[62] Morris C.J., Yang J.N., Garcia J.I., Myers S., Bozzi I., Wang W., Buxton O.M., Shea S.A., Scheer F.A. (2015). Endogenous circadian system and circadian misalignment impact glucose tolerance via separate mechanisms in humans. Proc. Natl. Acad. Sci. USA.

[63] EasyGV. https://www.phc.ox.ac.uk/research/technology-outputs/easygv.

[64] Van Cauter E., Polonsky K.S., Scheen A.J. (1997). Roles of circadian rhythmicity and sleep in human glucose regulation. Endocr. Rev..

[65] Hibi M., Masumoto A., Naito Y., Kiuchi K., Yoshimoto Y., Matsumoto M., Katashima M., Oka J., Ikemoto S. (2013). Nighttime snacking reduces whole body fat oxidation and increases LDL cholesterol in healthy young women. Am. J. Physiol. Regul. Integr. Comp. Physiol..

[66] Choi H.R., Kim J., Lim H., Park Y.K. (2018). Two-Week Exclusive Supplementation of Modified Ketogenic Nutrition Drink Reserves Lean Body Mass and Improves Blood Lipid Profile in Obese Adults: A Randomized Clinical Trial. Nutrients.

[67] Jakubowicz D., Wainstein J., Landau Z., Raz I., Ahren B., Chapnik N., Ganz T., Menaged M., Barnea M., Bar-Dayan Y. (2017). Influences of Breakfast on Clock Gene Expression and Postprandial Glycemia in Healthy Individuals and Individuals With Diabetes: A Randomized Clinical Trial. Diabetes Care.

[68] Rahman S., Islam R. (2011). Mammalian Sirt1: Insights on its biological functions. Cell Commun. Signal.

[69] Martinez-Lopez N., Tarabra E., Toledo M., Garcia-Macia M., Sahu S., Coletto L., Batista-Gonzalez A., Barzilai N., Pessin J.E., Schwartz G.J. (2017). System-wide Benefits of Intermeal Fasting by Autophagy. Cell Metab..

[70] Mani K., Javaheri A., Diwan A. (2018). Lysosomes Mediate Benefits of Intermittent Fasting in Cardiometabolic Disease: The Janitor Is the Undercover Boss. Compr. Physiol..

