# Supplementary material for: Early Time-Restricted Feeding Improves 24-Hour Glucose Levels and Affects Markers of the Circadian Clock, Aging, and Autophagy in Humans
Source: Nutrients. 2019 May 30;11(6):1234. doi: 10.3390/nu11061234 (PMC6627766; doi:10.3390/nu11061234)
Supplement: Supplementary file 1 [file nutrients-11-01234-s001.pdf]

nutrients

MDPI

SUPPLEMENTARY MATERIAL

Table S1: Genes and Their Accession Numbers

**Housekeeping**		
POLR2A	NM_000937.2	Homo sapiens polymerase (RNA) II (DNA directed) polypeptide A, 220 kDa
CDK4	NM_000075.2	Homo sapiens cyclin-dependent kinase 4
ACTB	NM_001101.2	Homo sapiens actin, beta
GAPDH	NM_002046.3	Homo sapiens glyceraldehyde-3-phosphate dehydrogenase
LDHA	NM_001165414.1	Homo sapiens lactate dehydrogenase A, transcript variant 3
TBP	NM_001172085.1	Homo sapiens TATA-box binding protein, transcript variant 2
**Glucose Uptake**		
SLC2A1 (GLUT1)	NM_006516.2	Homo sapiens solute carrier family 2 member 1
SCL2A4 (GLUT4)	NM_001042.2	Homo sapiens solute carrier family 2 member 4
AKT2	NM_001626.4	Homo sapiens AKT serine/threonine kinase 2, transcript variant 1
IRS1	NM_005544.2	Homo sapiens insulin receptor substrate 1
IRS2	NM_003749.2	Homo sapiens insulin receptor substrate 2
**Circadian System**		
BMAL1 (ARNTL)	NM_001030272.1	Homo sapiens aryl hydrocarbon receptor nuclear translocator-like, transcript variant 2
CLOCK	NM_004898.2	Homo sapiens clock homolog
PER1	NM_002616.2	Homo sapiens period circadian clock 1
PER2	NM_022817.2	Homo sapiens period circadian clock 2
CRY1	NM_004075.3	Homo sapiens cryptochrome 1 (photolyase-like)
CRY2	NM_001127457.1	Homo sapiens cryptochrome circadian clock 2, transcript variant 2
REV-ERBA (NR1D1)	NM_021724.3	Homo sapiens nuclear receptor subfamily 1, group D, member 1
RORA	NM_134261.2	Homo sapiens RAR related orphan receptor A, transcript variant 1
**Aging**		
SIRT1	NM_012238.4	Homo sapiens sirtuin 1, transcript variant 1
MTOR	NM_004958.3	Homo sapiens mechanistic target of rapamycin kinase
RICTOR	NM_152756.3	Homo sapiens RPTOR independent companion of MTOR, complex 2
RAPTOR	NM_020761.2	Homo sapiens regulatory associated protein of MTOR complex 1, transcript variant 1
**Autophagy**		
ATG5	NM_004849.2	Homo sapiens autophagy related 5
ATG7	NM_001136031.2	Homo sapiens autophagy related 7, transcript variant 2
ATG12	NM_004707.3	Homo sapiens autophagy related 12, transcript variant 1
LC3A	NM_032514.2	Homo sapiens microtubule-associated protein 1 light chain 3 alpha, transcript variant 1
LAMP2	NM_002294.2	Homo sapiens lysosomal associated membrane protein 2, transcript variant A
**Oxidative Stress**		
SOD1	NM_000454.4	Homo sapiens superoxide dismutase 1
SOD2	NM_000636.2	Homo sapiens superoxide dismutase 2, transcript variant 1
GPX3	NM_002084.3	Homo sapiens glutathione peroxidase 3
NOX1	NM_007052.4	Homo sapiens NADPH oxidase 1, transcript variant 1
NOS3	NM_000603.4	Homo sapiens nitric oxide synthase 3, transcript variant 1
